# Extra-Peritoneal Lipoma

**Published:** 1896-01

**Authors:** W. H. Wathen

**Affiliations:** Professor of Abdominal Surgery and Gynæcology in the Kentucky School of Medicine; Fellow of the American Gynæcological Society and of the Southern Surgical and Gynæcological Society; Gynæcologist to the Kentucky School of Medicine Hospital and the Louisville City Hospital, Louisville, Kentucky


### EXTRA-PERITONEAL LIPOMA.

REPORTED TO THE LOUISVILLE CLINICAL SOCIETY.

BY W. H. WATHEN, A. M., M. D.,

Professor of Abdominal Surgery and Gynæcology in the Kentucky School of Medicine; Fellow of the American Gynæcological Society and of the Southern Surgical and Gynæcological Society; Gynæcologist to the Kentucky School of Medicine Hospital and the Louisville City Hospital, Louisville, Kentucky.

A lady from Indiana came to this city a few days ago to consult me, and upon examination, a tumor could be distinctly outlined, beginning in the region of the kidney posteriorly, extending down to the inguinal region on the left side. The abdomen was very fat, the woman being large, and when the tumor was brought between the hands it would slip out, just as is observed in cases of movable or floating kidney, but it extended so low that I could not believe it was a kidney, though so movable that it appeared to be in the abdominal cavity. I suspected that it was connected with the ovary by a long pedicle.

An operation was advised and accepted. With one stroke of the scalpel, the tumor was exposed, and was found to be a solid growth, which was extra-peritoneal and easily enucleated. When the tumor was taken away, there was no opening in the peritoneal cavity. There was no pedicle to ligate and no hæmorrhage. The tumor has the appearance of being an enlarged kidney, but I am unable to determine the character of the growth unless it be subperitoneal lipoma, as is sometimes found in the abdominal walls of fat women. Possibly it may be a sarcoma, but a sarcoma could hardly have been so well defined and so easily separated from adjacent structures. Dr. H. H. Koehler has made sections of the tumor for microscopical examination. No shock followed the operation, of course; there is no reason why there should have been any; the woman continued in a normal condition, with pulse 65 to 70, and no elevation of temperature. Bowels moved regularly, and she ate and slept well for five days. On Tuesday afternoon, six days after the operation, she was lying on her side, talking to her daughter; she suddenly turned on her back and, in an instant, showed that the heart's action had nearly stopped. I reached her fifteen minutes later and found her nearly pulseless, with cold, clammy perspiration, and severe pain in the region of the heart. Under the use of heart stimulants, she rallied, the pulse becoming 115 to 120 to the minute and very good volume. She then told me that she had suffered such "spells" on many previous occasions. I left her, giving instructions to the nurse what to do in case there was a recurrence of the trouble. She continued in good condition for perhaps thirty minutes, and then, the nurse reports that within less than half a minute she was dead. No post-mortem examination was performed.

It seems that this patient, for a number of years, had suffered from some form of organic heart trouble, although Dr. Guest, who administered the chloroform, examined her heart and did not detect any serious trouble. There was no trouble from the anæsthesia; the pulse was of good volume throughout the operation and remained so for five days afterward.

